# NAD+ improves neuromuscular development in a zebrafish model of FKRP-associated dystroglycanopathy

**DOI:** 10.1186/s13395-019-0206-1

**Published:** 2019-08-07

**Authors:** Erin C. Bailey, Sarah S. Alrowaished, Elisabeth A. Kilroy, Emma S. Crooks, Daisy M. Drinkert, Chaya M. Karunasiri, Joseph J. Belanger, Andre Khalil, Joshua B. Kelley, Clarissa A. Henry

**Affiliations:** 1 School of Biology and Ecology, University of Maine, Orono, ME 04469 USA; 2 Chemical and Biomedical Engineering, University of Maine, Orono, ME 04469 USA; 3 Molecular and Biomedical Sciences, University of Maine, Orono, ME 04469 USA; 4 Graduate School of Biomedical Sciences and Engineering, University of Maine, 217 Hitchner Hall, Orono, ME 04469 USA; 5 Present Address: Tufts University School of Medicine, Boston, MA 02111 USA; 6 Present Address: Lake Erie College of Osteopathic Medicine, Erie, PA 16509 USA

**Keywords:** Zebrafish, Dystroglycanopathy, FKRP, NAD+, Neuromuscular junction

## Abstract

**Background:**

Secondary dystroglycanopathies are muscular dystrophies that result from mutations in genes that participate in Dystroglycan glycosylation. Glycosylation of Dystroglycan is essential for muscle fibers to adhere to the muscle extracellular matrix (myomatrix). Although the myomatrix is disrupted in a number of secondary dystroglycanopathies, it is unknown whether improving the myomatrix is beneficial for these conditions. We previously determined that either NAD+ supplementation or overexpression of Paxillin are sufficient to improve muscle structure and the myomatrix in a zebrafish model of primary dystroglycanopathy. Here, we investigate how these modulations affect neuromuscular phenotypes in zebrafish fukutin-related protein (*fkrp*) morphants modeling FKRP-associated secondary dystroglycanopathy.

**Results:**

We found that NAD+ supplementation prior to muscle development improved muscle structure, myotendinous junction structure, and muscle function in *fkrp* morphants. However, Paxillin overexpression did not improve any of these parameters in *fkrp* morphants. As movement also requires neuromuscular junction formation, we examined early neuromuscular junction development in *fkrp* morphants. The length of neuromuscular junctions was disrupted in *fkrp* morphants. NAD+ supplementation prior to neuromuscular junction development improved length. We investigated NMJ formation in *dystroglycan* (*dag1*) morphants and found that although NMJ morphology is disrupted in *dag1* morphants, NAD+ is not sufficient to improve NMJ morphology in *dag1* morphants. Ubiquitous overexpression of Fkrp rescued the *fkrp* morphant phenotype but muscle-specific overexpression only improved myotendinous junction structure.

**Conclusions:**

These data indicate that Fkrp plays an early and essential role in muscle, myotendinous junction, and neuromuscular junction development. These data also indicate that, at least in the zebrafish model, FKRP-associated dystroglycanopathy does not exactly phenocopy DG-deficiency. Paxillin overexpression improves muscle structure in *dag1* morphants but not *fkrp* morphants. In contrast, NAD+ supplementation improves NMJ morphology in *fkrp* morphants but not *dag1* morphants. Finally, these data show that muscle-specific expression of Fkrp is insufficient to rescue muscle development and homeostasis.

**Electronic supplementary material:**

The online version of this article (10.1186/s13395-019-0206-1) contains supplementary material, which is available to authorized users.

## Background

Muscle is a highly conserved tissue that is part of the neuromusculoskeletal system and is essential for strength, locomotion, and health. The neuromuscular junction (NMJ) initiates muscle contraction, whereas the myotendinous junction (MTJ) is the major site of force transmission from muscle to the skeletal system. Muscle fibers adhere to their surrounding extracellular matrix (ECM), the MTJ, and the NMJ; and these adhesion complexes are critical for muscle development and homeostasis (reviewed in [26]). The muscle extracellular matrix and adhesion complexes at MTJs and NMJs are specialized and allow the muscle to adhere to and interact with neurons and tendons during development and homeostasis. Thus, regulation of cell-ECM adhesion is essential for development and homeostasis of the neuromusculoskeletal system.

The dystrophin-glycoprotein complex (DGC) is an essential component of muscle-ECM adhesion. The DGC is a multi-protein complex that indirectly links the actin cytoskeleton of muscle fibers to the laminin-rich basement membrane in the ECM [18]. The DGC is thought to provide mechanical stabilization during muscle contraction [18, 60] in addition to its role as a signaling complex [12]. Different variants of the DGC are present at the MTJ versus the NMJ. Whereas Dystrophin is the predominant protein that connects the actin cytoskeleton to the transmembrane protein Dystroglycan (DG) at the MTJ [18], Utrophin mainly establishes this connection at the NMJ [16]. Despite these differences, DG is present and highly glycosylated at both the MTJ and the NMJ. DG glycosylation is required for DG binding to basement membrane proteins such as laminin, agrin, and perlecan [8, 20, 49]. Dystroglycanopathies result from mutations in genes responsible for glycosylation of alpha-DG (DG contains two subunits: a transmembrane beta subunit and an extracellular alpha subunit) [23, 33, 51]. Thus far, 16 gene products that participate in DG glycosylation have been identified [52]. In addition to muscle degeneration, dystroglycanopathies are frequently associated with central nervous system defects such as intellectual disability and brain malformation [52]. The role of DG glycosylation in the peripheral nervous system is less understood although abnormal NMJs have been reported after birth in a couple of dystroglycanopathy mouse models [13, 32, 43, 54, 61]. Because NMJ defects have been observed after embryonic development, the most prevalent hypothesis is that post-synaptic DG does not play a role in NMJ formation, but is important for NMJ stabilization [52]. For example, patients with mutations in *GMPPB* (required for GDP-mannose formation [36]) show decreased action potentials with repeated nerve stimulation [37]. Given the plethora of glycosylated proteins at the NMJ, it is possible that a subset of dystroglycanopathy genes may be required outside of muscle tissue for NMJ development.

FKRP-associated dystroglycanopathy results from mutations in *FKRP*, which encodes an enzyme critical for DG glycosylation. FKRP works in concert with Fukutin to transfer CDP-ribitol synthesized by Isoprenoid Synthase Domain-Containing (ISPD) protein to alpha-DG [21, 38] to form a tandem ribitol-5-phosphate that links xylose with *N*-acetylgalactosamine [69]. This process is necessary for DG glycosylation, which is important for DG binding to basement membrane proteins [8, 20, 49]. Patients with FKRP-associated dystroglycanopathy present with phenotypic variability; however, muscle weakness and elevated serum creatine kinase are consistently present [37]. Depending on the molecular basis of the *FKRP* mutation, individuals with this condition may develop limb-girdle muscular dystrophy 2I (LGMD2I) or congenital muscular dystrophy with or without eye and brain involvement [67, 75]. There is some genotype-phenotype correlation in patients with C-terminal *FKRP* mutations tending to be more severe [4, 6, 67, 75]. However, there is remarkable phenotypic variation among individuals with the same mutation [67]. For example, a study of 25 patients homozygous for c826C/A found significant variation in muscle pathology, and levels of glycosylated DG [67]. Neither the histopathological alterations nor levels of DG glycosylation correlated with age of onset or walking function. Interestingly, whereas one set of siblings presented with similar clinical and histopathological features, a second set of siblings had dramatic variation in the age of onset (12 vs 27 years old) [67]. These data suggest that there are modifying factors—environmental and/or genetic—that affect progression of FKRP-associated dystroglycanopathy. These data also suggest that it is imperative to study features other than muscle pathology and/or levels of DG glycosylation. Mouse [1] and zebrafish models of FKRP deficiency have been used to study mechanisms associated with FKRP-associated dystroglycanopathy. Zebrafish models of FKRP-associated dystroglycanopathy include morpholino (MO) models [39, 72] and a more recently generated *fkrp* mutant [63]. These studies indicate that the *fkrp* morphants phenocopy the *fkrp* mutant, that *fkrp* deficiency results in impaired muscle development and wasting, and that laminin deposition at the MTJ is disrupted in Fkrp-deficient zebrafish.

Laminin is a major component of the basement membrane that surrounds muscle. Laminin is a heterotrimeric protein with an alpha, beta, and gamma chain. Adhesion of muscle fibers to laminin is necessary for muscle development and homeostasis [2, 28, 29, 64, 65, 80]. The principle laminin isoform in mature vertebrate skeletal muscle is laminin-211 (alpha2 beta1 gamma1). Mutations in the human *laminin alpha2* gene result in MDC1A, a common congenital muscular dystrophy [31]. A different laminin isoform, laminin-111, is the major isoform expressed during muscle development, and laminin-111 is required for muscle development in mouse and zebrafish [2, 65]. Laminin-111 can partially compensate for laminin-211. Overexpression of laminin alpha1 slows the progression of dystrophy in *laminin alpha2* mutant mice [19, 57]. Injection of laminin-111 protein directly into muscle of *mdx* mice modeling Duchenne muscular dystrophy (DMD) improves muscle structure and function [56]. These data indicate that understanding mechanisms that mediate laminin-111 expression and polymerization during muscle development could provide information for future therapeutic development. We identified a novel pathway required for laminin-111 organization at the zebrafish MTJ during muscle development. Nicotinamide Riboside Kinase 2b (previously called muscle Integrin binding protein) [44, 45] is necessary for normal laminin-111 organization [24]. Yeast and human Nrk2s function in an alternative salvage pathway that generates Nicotinamide Adenine Dinucleotide (NAD+) [7, 70]. Exogenous NAD+ rescues MTJ morphogenesis in Nrk2b-deficient zebrafish, indicating that zebrafish Nrk2b also functions to generate NAD+ [24]. Given that NAD+ biosynthesis is necessary for normal laminin-111 organization during muscle development, we asked whether exogenous NAD+ would be sufficient to improve muscle structure and function in zebrafish modeling muscular dystrophies. NAD+ supplementation increases laminin organization and reduces muscle degeneration in zebrafish deficient for either of the laminin-211 receptors (DG or Integrin alpha7 (Itga7)) [25]. As vitamin B3 is a precursor for NAD+, we asked whether vitamin supplementation would increase NAD+ and improve muscle structure and function in DG-deficient zebrafish. Supplementation with EmergenC packets that contain B vitamins (chosen because they are water soluble) improves muscle structure and motility in DG-deficient zebrafish.

The two major transmembrane receptors that anchor muscle cells to laminin in their ECM are DG (described above) [18, 20] and Integrin alpha7 (Itga7) [11, 66]. Itga7 is also required for muscle homeostasis: mutations in *Itga7* lead to congenital muscular dystrophy with Itga7 deficiency [30]. These two cell adhesion complexes display some degree of functional redundancy in both zebrafish and mouse models. Pharmacologically increasing levels of Itga7 compensates for the loss of Dystrophin in a mouse model of DMD [62, 76]. Given that NAD+ is sufficient to improve muscle structure in zebrafish deficient for either laminin receptor complex (DG or Itga7), we hypothesized that NAD+ increases laminin organization by increasing clustering of the remaining receptor [25]. Our model is that in the absence of Itga7, NAD+ increases DG clustering, thus improving muscle-ECM adhesion. Similarly, we hypothesize that in the absence of DG, NAD+ increases Itga7 clustering, thus improving muscle-ECM adhesion. This model leads to the question of whether, if DG is present but hypoglycosylated, NAD+ would be sufficient to improve muscle structure in a zebrafish secondary dystroglycanopathy model. Our previous data showed beneficial effects of NAD+ when administered prior to muscle development [25]. Whether NAD+ is beneficial after initial muscle development in this context has not yet been determined.

Paxillin is an Integrin-associated adaptor protein that concentrates at the MTJ during muscle development [14]. Paxillin is an essential signaling nexus that regulates cell adhesion, morphology, and migration [15]. Paxillin participates in the Nrk2b-laminin pathway. Nrk2b is cell-autonomously required for subcellular concentration of Paxillin at the MTJ, and Paxillin overexpression rescues muscle development Nrk2b-deficient zebrafish [24]. DG is required for normal Paxillin concentration at the MTJ: DG-deficient zebrafish show reduced concentration of Paxillin at the MTJ. Addition of NAD+ improves Paxillin concentration at the MTJ and Paxillin overexpression reduces muscle degeneration in DG-deficient zebrafish [25]. In contrast, Paxillin overexpression does not rescue zebrafish deficient for Itga7 [25]. These data suggest that Paxillin functions downstream of NAD+ to improve muscle resilience in DG-deficient zebrafish. However, whether Paxillin concentration at the MTJ is disrupted in zebrafish models of secondary dystroglycanopathy and whether Paxillin overexpression ameliorates muscle degeneration in zebrafish models of secondary dystroglycanopathy have not been investigated.

We used the *fkrp* morphant model of FKRP-associated dystroglycanopathy [39, 72] to address unanswered questions regarding NAD+ regulation of the ECM in a secondary dystroglycanopathy. In addition, given the data indicating that there is not a strict correlation between DG glycosylation levels and phenotype, we investigated neuromuscular junction development in *fkrp* morphants. Supplementing *fkrp* morphants with NAD+ at gastrulation improves laminin polymerization at the MTJ, muscle structure, and muscle function. Despite the fact that Paxillin localization is disrupted in *fkrp* morphants, Paxillin overexpression failed to rescue any of these phenotypes. Early NMJ development was disrupted in *fkrp* morphants and improved with NAD+. To our knowledge, this is the first report of initial NMJ formation being disrupted in an animal model of secondary dystroglycanopathies. As initial NMJ development has not been investigated in an animal model of DG deficiency, we analyzed early NMJ development in *dag1* morphants. NMJ development is disrupted in *dag1* morphants, although to a lesser extent than *fkrp* morphants. In contrast to *fkrp* morphants, NAD+ supplementation did not improve NMJ development in *dag1* morphants. Finally, we show that muscle-specific overexpression of *fkrp* improved MTJ morphology but was not sufficient to improve muscle structure or function. Taken together, these data indicate that, at least in the zebrafish, FKRP-associated dystroglycanopathy does not phenocopy DG-deficiency. Furthermore, these data show that Fkrp is required for normal NMJ development and is required in tissues other than muscle.

## Methods

### Zebrafish husbandry and transgenic lines

All embryos were obtained from natural spawnings of adult zebrafish maintained on a 14-h light/10-h dark cycle at 28.5 °C. Embryos were reared in 1X embryo rearing medium (ERM) with methylene blue and staged according to [40]. The following strains were utilized/generated: AB, *Tg* (*fli1*:*EGFP*) [42], *Tg* (*ef1α*:*xbp1δ*-*GFP*) [46] (a kind gift from Dr. Shao Jun Du), *Tg* (*hsp70l*:*pxn*-*EGFP*) [10], *Tg* (*hsp70l*:*fkrp*-*EGFP*), and *Tg*(-*503unc*:*fkrp*-*EGFP*). The *Tg* (*hsp70l*:*fkrp*-*EGFP*) and *Tg*(*-503unc*:*fkrp*-*EGFP*) lines were generated via the gateway cloning system. A sequence surrounding the *fkrp* gene was amplified with NCBI-verified primers and was gated and inserted into the pDONR221 vector (Invitrogen). The heat shock gateway 222 5′ vector, GFP gateway 366 3′ vector, and the gateway 394 destination vector [41] were used along with the donor vector and LR clonase II for the *Tg* (*hsp70l*:*fkrp*-*EGFP*) line. Components remained the same for the *Tg*(*-503unc*:*fkrp*-*EGFP*) line with the exception of the -*503unc* zebrafish muscle-specific promoter [5], which was used in place of the heat shock gateway 222 5′ vector. Plasmids were injected at the single cell stage as previously described [24].

### MO injections

Antisense MOs were obtained from Gene Tools, LLC, and hydrated at 65 °C for 10 min with sterile water to generate 1-mM stocks. For *fkrp* morphant experiments, the previously published *fkrp* MO2 (5′-CTTGTGGTTTTATGGCAGAAAGAGT-3′) [39] was utilized and injected into the yolk of 1–2 cell stage embryos so that embryos received approximately 3.2 ng of MO. For *dag1* morphant experiments, the previously published *dag1* MO (*dag1* MO1) (5′-CATGCCTGCTTTTATTTTCCCTCGC-3′ [53] and an additional slightly overlapping *dag1* MO (*dag1* MO2) (5′-CCCTCGCTCGTACAAAAGAGGACGT-3′) were co-injected into the yolk of 1–2 cell stage embryos so that embryos received approximately 12 ng of each *dag1* MO1 and *dag1* MO2. Embryos utilized in experiments alongside NAD+- and EmergenC-treated embryos were separated into 60-mm Petri dishes with 25 embryos per dish in 10 mL 1X ERM.

### NAD+ and EmergenC supplementation

*Fkrp* morphants receiving EmergenC treatment (Alacer Corp) were separated into 60-mm Petri dishes with 25 embryos per dish in 10 mL 1X ERM-EmergenC solution. The NAD+ solution was prepared as previously described [24] except in sterile water. The EmergenC solution was diluted in 1X ERM so that the level of niacin in the solution was equal to 7.61 μM. Supplementation began at 6 h post fertilization (hpf) or 24 hpf depending on the experiment, and the solution was changed every 24 h until embryo fixation.

### Expression of constructs with the heat shock promoter

To constitutively overexpress Fkrp, uninjected and injected *Tg* (*hsp70l*:*fkrp*-*EGFP*) embryos were heat shocked at 38 °C for 1.25–2 h at the 15 somite stage. To overexpress Paxillin, *Tg* (*hsp70l*:*pxn*-*EGFP*) were heat shocked at 38 °C for 1.5 h at 12 hpf, then for 1.5–2 h daily until fixation. Heat-shocked fish were immediately transferred to 28 °C.

### Mobility assays

Touch response analysis was performed at 3 dpf. Larvae were placed in 1X ERM and stimulated at the posterior end with fishing wire. The number of touches to evoke an escape response was recorded. For larvae that did not respond after 50 touches, 50 was recorded as their touches to response.

### Phalloidin staining, bungarotoxin staining, and immunohistochemistry

All embryos were fixed in 4% Paraformaldehyde (PFA) for 2–4 h at room temperature, depending on the stage. After fixation, embryos received five rinses in PBS-0.1% Tween 20. Unless co-stained with alpha-bungarotoxin, embryos stained with phalloidin were permeabilized for 1.5 h in PBS-2% Triton-X-100, followed by incubation in 1/20 phalloidin-546 (Invitrogen) in PBS-2% Triton-X-100 or PBS-0.1% Tween 20 overnight at 4 °C. Embryos stained with alpha-bungarotoxin (Molecular Probes) and SV2 were permeabilized in 1 mg/ml collagenase in 1X PBS for 75 min, then were stained with 1/500 alpha-bungarotoxin-647 for 2–4 h at room temperature. For co-staining, 1/20 phalloidin-546 was added to the alpha-bungarotoxin solution in antibody block. All embryos were then subjected to five rinses for 5 min each in PBS-0.1% Tween 20, followed by an overnight incubation in antibody block (5% BSA, 1% DMSO, 1% Triton-X-100, 0.2% saponin in 1X PBS) for embryos receiving subsequent immunohistochemistry treatments. Primary antibodies were added at a concentration of 1/50 (anti-laminin (Thermo Scientific), anti-SV2 (Developmental Studies Hybridoma Bank), anti-Paxillin (BD Biosciences), anti-beta-DG (Novocastra-Leica)) in antibody block and incubated for 2–8 h at room temperature followed by overnight at 4 °C. Embryos treated with anti-SV2 were incubated for 3 days at 4 °C following the initial overnight incubation. All embryos were then treated with antibody block for 8 h at room temperature or overnight at 4 °C, followed by an overnight incubation 4 °C plus 2–6 h at room temperature in secondary antibody (Invitrogen) diluted 1/150–1/200 in block. Embryos were rinsed out of secondary antibody with PBS-0.1% Tween 20 prior to deyolking, mounting, and imaging.

### Imaging

Fixed and stained embryos were deyolked, then mounted and imaged in 80% glycerol. Fluorescent images of fixed, stained fish were captured at × 20 with an Olympus Fluoview IX-81 inverted microscope with FV1000 confocal system or at × 25 with a Leica DMi8 confocal microscope. Images for *Tg* (*ef1α*:*xbp1δ*-*GFP*) were acquired at × 10 on a Zeiss Axio Imager Z1 microscope with a Zeiss ApoTome or at × 5 on a Zeiss microscope with Vivatome attachment. Exposure time was kept consistent between all embryo groups within an experiment.

### Statistical analysis

All data were log transformed in GraphPad PRISM 8 before performing statistical comparisons. All subsequent analyses were performed in GraphPad PRISM 8. Statistical comparisons of data were performed using a two-tailed student *t* test for comparisons of two groups, and an ordinary one-way ANOVA with Tukey’s ad hoc analysis for assays with three or more groups. Categorical data were scored in using Fisher’s exact test for two categories and the chi square test for three or more categories.

### Image analysis

Muscle fiber degeneration was quantified by counting the number of muscle segments with degeneration per embryo and calculating the percent of myotomes with muscle degeneration. MTJ angles were analyzed in FIJI by using the angle tool to measure the angles formed by the MTJ. For laminin and beta-DG staining analyses, images were blinded using a Perl script and embryos were scored according to their relative staining intensity (Additional file 1: Figure S1). Intersegmental vessel (ISV) lengths were measured in FIJI using the segmented line tool.

For anisotropy analysis, a polygon was drawn in eight myotomes per image analyzed using the polygonal lasso tool in Adobe Photoshop. The *x*,*y* coordinates of each polygon were recorded. Masks were generated from the coordinates, and 2DWTMM analysis was performed on each of these masks as previously described [24, 25].

For analysis of *xbp1* transgenic embryos, fluorescence intensity was quantified using FIJI. A polygon was drawn around the dorsal muscle above the notochord for all embryos in the trunk region. The average pixel intensity (mean gray value) was calculated. The mean average pixel intensity for all controls was calculated. These values were used to determine the percent average pixel intensity for all embryos. Images for all *xbp1* transgenic embryos were blinded using a Perl script prior to analysis.

For NMJ analyses, confocal images were processed into maximum intensity projections of fast-twitch (distributed) and slow-twitch (myoseptal) innervation using FIJI and were subsequently blinded using a Perl script. Masks for the fish, horizontal myoseptum, and myoseptal innervation were generated using FIJI. The masks were imported into MATLAB (Mathworks) and used to segment acetylcholine receptor (AChR) and Synaptic vesicle protein 2 (SV2) fluorescence channels. The fluorescence images were enhanced using adaptive histogram equalization (MATLAB “adapthisteq” function) and then denoised with a 1-pixel radius gaussian filter (MATLAB “imguassfilt” function). Single masks of the fluorescence channels were then generated using a threshold of 30 on each channel, which were then combined with an “or” statement. Thresholds up to 58 and down to 10 yielded the same trend in the data, indicating that the measurements chosen were not overly sensitive to this parameter. The masks were skeletonized, and subsequently cleaned and despurred using the MATLAB “bwmorph” command. Branchpoints were also identified using the MATLAB “bwmorph” command. The fish, horizontal myoseptum, and myoseptal innervation masks were used to define the muscle segments, and the indicated measurements were performed for each muscle segment across all embryos analyzed.

## Results

### NAD+ supplementation prior to muscle development improves muscle and MTJ structure

**Fig. 1:**
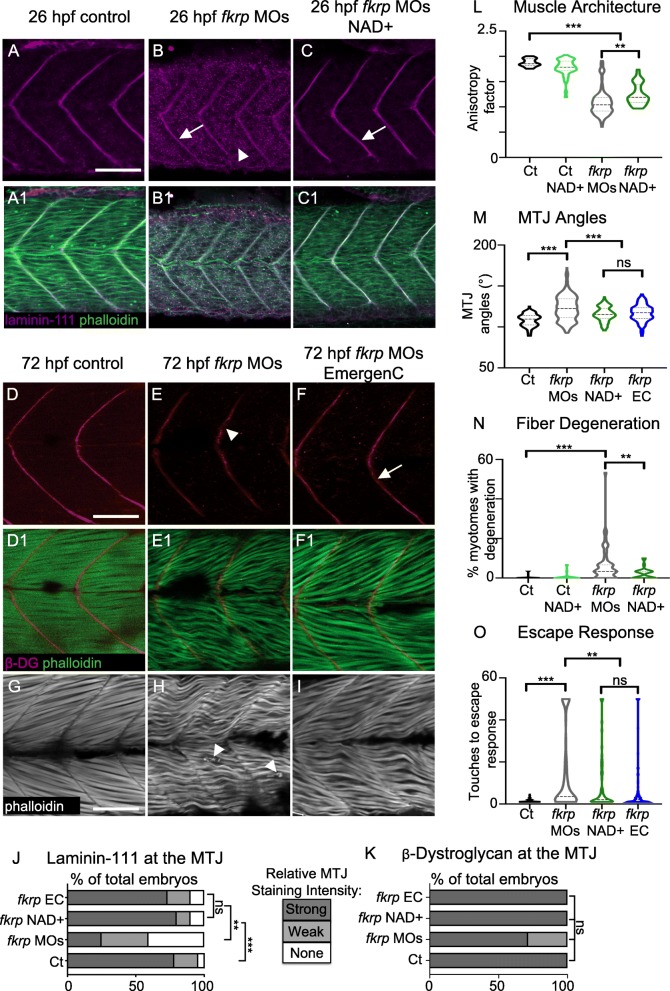
NAD+ or EmergenC supplementation at gastrulation improves muscle structure and function in *fkrp* morphants. (**A–C**) Anterior left, dorsal top, side-mounted embryos at 26 hpf stained for laminin-111 (purple) and actin (phalloidin, green). (**A, A1**) Control embryo. Laminin is concentrated at the MTJ. (**B, B1**) *fkrp* morphant. Although laminin is present at the MTJ (white arrow), it is also present within the myotome (white arrowhead). (**C, C1**) *fkrp* morphant treated with 0.1 mM NAD+ at 6 hpf. Laminin is concentrated at the MTJ (white arrow) as in control embryos. (**D–F1**) Anterior left, dorsal top, side-mounted embryos at 72 hpf stained for beta-DG (red) and f-actin (phalloidin, green). (**D, D1**) Control embryo. (**E, E1**) *fkrp* morphant. Beta-DG staining appears weaker at the MTJ (white arrowhead). (**F, F1**) *fkrp* morphant treated with EmergenC at 6 hpf. Beta-DG staining appears stronger at the MTJ (white arrow). (**G–I**) Anterior left, dorsal top, side-mounted embryos at 72 hpf stained for f-actin (phalloidin, gray). (**G**) Control embryo. (H) *fkrp* morphant. White arrowheads indicate single detached fibers. (**I**) *fkrp* morphant treated with EmergenC at 6 hpf. Note the improved muscle fiber structure. (**J**) Relative staining intensity of laminin-111 at the MTJ in 26 hpf embryos, based on no staining/localization (none, white), weak staining/localization (weak, light gray), and strong staining/localization (strong, dark gray) (images were blinded prior to analysis, see the “Methods” section). Controls (*n* = 23 embryos) have greater laminin intensity staining than *fkrp* morphants (*n* = 32 embryos). NAD+ (*n* = 10 embryos) and EmergenC (*n* = 30 embryos) supplementation improve laminin-111 concentration at the MTJ in *fkrp* morphants. (**K**) Relative staining intensity of beta-DG at the MTJ in 72 hpf embryos, based on weak staining (weak, light gray) and strong staining (strong, dark gray). Although *fkrp* morphants have more embryos with weaker beta-DG staining at the MTJ (*n* = 7 embryos), there is no significant difference in beta-DG at the MTJ between untreated morphants, morphants treated with NAD+ (*n* = 7 embryos), EmergenC (*n* = 7 embryos), or control morphants (*n* = 5 embryos). (**L**) Quantification of muscle organization at 72 hpf. The anisotropy factor in embryos injected with *fkrp* MOs (*n* = 96 half-myotomes) is reduced compared to uninjected controls (*n* = 16 half-myotomes), and NAD+ (*n* = 40 half-myotomes) significantly increases the anisotropy factor in *fkrp* morphants. (**M**) Quantification of MTJ angles at 72 hpf. Injection of *fkrp* MOs (*n* = 343 MTJs) significantly increases MTJ angles compared to uninjected controls (*n* = 98 MTJs). Either NAD+ (*n* = 105 MTJs) or EmergenC (*n* = 158 MTJs) treatment significantly reduces MTJ angles compared to untreated morphants. (**N**) Untreated *fkrp* morphants (*n* = 54 embryos) have a significantly higher percent of myotomes per embryo with fiber detachments than controls (*n* = 40 embryos) at 72 hpf. NAD+ (*n* = 31 embryos) supplementation significantly reduces the percent of myotomes with dystrophy per embryo. (**O**) Injection of *fkrp* MOs (*n* = 184 embryos) significantly increases the number of touches required to induce an escape response compared to uninjected controls (*n* = 185 embryos). NAD+ (*n* = 84 embryos) or EmergenC (*n* = 111 embryos) treatment significantly reduces the number of touches to invoke an escape response. Scalebars are 50 μm. *p < 0.05, **p < 0.01, ***p < 0.001, ns non-significant

Injection of previously published *fkrp* MOs recapitulated the previously described phenotype of *fkrp* morphants [39, 72] and the recently described *fkrp* mutant [63]. In agreement with previous findings, multiple aspects of muscle development are disrupted in *fkrp* morphants: MTJs are significantly wider than in control embryos (Fig. 1M), muscle fibers are disorganized (Fig. 1L), and muscle degeneration is observed (Fig. 1H, N). Muscle fiber adhesion to the MTJ and ECM organization at the MTJ are disrupted in *fkrp* morphants [39, 72]. We also found that laminin (Fig. 1A, B, J) is reduced in *fkrp* morphants compared to controls and that beta-DG is concentrated at the MTJ, although sometimes slightly reduced (Fig. 1D, E, K). We next asked whether muscle function was disrupted by quantifying the number of touches required to induce an escape response. We found that *fkrp* morphants have reduced muscle function in that they require significantly more touches to induce an escape response (Fig. 1O). These data indicate that *fkrp* is necessary for normal muscle development and function.

We previously showed that either NAD+ or EmergenC supplementation at gastrulation is sufficient to improve ECM organization, reduce muscle degeneration, and improve motility in *dag1* morphants [25]. We hypothesized that NAD+/EmergenC increased laminin organization by potentiating clustering of the other major laminin receptors in muscles, Integrins alpha6/beta1 and alpha7/beta1. We asked if NAD+/EmergenC is sufficient to improve laminin organization in the context of impaired DG glycosylation because hypoglycosylated DG could hinder increased laminin organization. NAD+ or EmergenC supplementation at gastrulation increased the concentration of laminin at the MTJ (Fig. 1C, J), decreased MTJ angles (Fig. 1M), decreased muscle degeneration (Fig. 1N), improved muscle fiber organization (Fig. 1L), and significantly reduced the touches required to evoke an escape response (Fig. 1O). These data indicate that NAD+ or EmergenC supplementation during gastrulation is sufficient to improve muscle development and function in Fkrp-deficient zebrafish.

### NAD+/EmergenC are not sufficient to improve abnormal vascularization, midbrain-hindbrain development, or the increased unfolded protein response in fkrp morphants

**Fig. 2:**
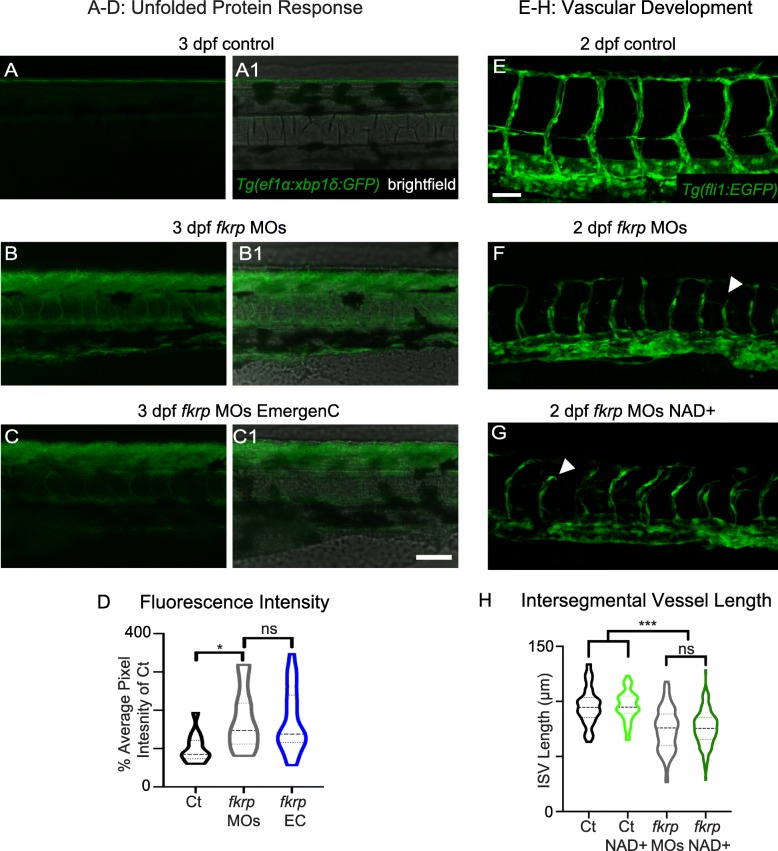
NAD+ and EmergenC supplementation at gastrulation do not significantly improve the UPR or vascularization in *fkrp* morphants. (**A–C1**) Anterior left, dorsal top, side-mounted 3 dpf embryos expressing *Tg* (*ef1α*:*xbp1δ*-*GFP*). Fluorescence intensity was kept constant within an experiment (see methods). Numbered panels are merged with the brightfield channel. (**A, A1**) Control embryo. Note the low relative expression of Xbp1 compared to morphants. (**B, B1**) *fkrp* morphant. (**C, C1**) *fkrp* morphant treated with EmergenC at gastrulation. Note that fluorescence intensity is similar to that of untreated morphant. (**D**) Quantification of Xbp1 fluorescence intensity as a percent of the wild-type value for all groups imaged. Fluorescence intensity is significantly increased in morphants. There is no significant difference in fluorescence intensity between untreated morphants (*n* = 15 embryos) and morphants receiving EmergenC (*n* = 21 embryos). (**E–G**) Anterior left, dorsal top, side-mounted 2 dpf embryos expressing *Tg* (*fli1*:*EGFP*) focused on the ISVs. (**E**) Control embryo. (F) *fkrp* morphant embryo. Note that some ISVs are truncated (white arrowhead). (**G**) *fkrp* morphant embryo treated with NAD+ at gastrulation. Truncated ISVs are still present in NAD+ treated morphants (white arrowhead). (**H**) Quantification of ISV length. ISV length is reduced in *fkrp* morphants (*n* = 201 vessels) compared with uninjected controls (*n* = 84 vessels). NAD+ supplementation (*n* = 166 vessels) does not rescue ISV length in *fkrp* morphants. Scalebars are 50 μm. *p < 0.05, **p < 0.01, ***p < 0.001, ns non-significant

Fkrp is required for normal zebrafish vascularization: *fkrp* morphants and the recently described *fkrp* mutant exhibit shorter intersegmental vessel (ISV) lengths compared to wild-type embryos [63, 78]. *Fli1*:*EGFP* transgenics injected with *fkrp* MOs exhibited shorter ISV lengths (including truncated vessels) than uninjected controls (Fig. 2E, F, H). NAD+ supplementation had no significant effect on ISV length in *fkrp* morphants (Fig. 2G, H). We also analyzed ISV length when normalized to the width of the embryos because muscle development is disrupted in *fkrp* morphants. The same result was observed with normalized data (not shown). These data suggest that: (1) NAD+ supplementation is not sufficient to improve vascularization in *fkrp* morphants, and (2) the vascularization defects observed in *fkrp* morphants are likely a consequence of *fkrp* knockdown and not aberrant muscle development and/or homeostasis.

Zebrafish deficient for Fkrp exhibit abnormal midbrain-hindbrain boundary formation that has been likened to cobblestone lissencephaly [72]. We also observed abnormal midbrain-hindbrain boundary formation in *fkrp* morphants at 26 hpf (*n* = 8) compared with uninjected controls. NAD+ was not sufficient to rescue midbrain-hindbrain boundary formation (*n* = 8, data not shown). Zebrafish models of FKRP-associated dystroglycanopathy are also associated with endoplasmic reticulum (ER) stress and activation of the unfolded protein response (UPR) [47]. One marker of the UPR is increased activation of the *xbp1* transcript, which is upregulated in *fkrp* morphants [47] and the recently described *fkrp* mutant [63]. To determine whether EmergenC could improve ER stress in *fkrp* morphants, we injected *fkrp* MOs into the *Tg* (*ef1α*:*xbp1δ*-*GFP*) line that allows visualization of *xbp1* activation [46]. Injection of *fkrp* MOs increased the UPR (Fig. 2B) compared with controls (Fig. 2A). However, untreated and EmergenC-treated *fkrp* morphants did not significantly differ in relative fluorescence intensity (Fig. 2C, D). Taken together, these results suggest that the benefits of NAD+ supplementation may be limited to muscle in Fkrp-deficient zebrafish.

### Paxillin overexpression does not significantly reduce muscle degeneration

**Fig. 3:**
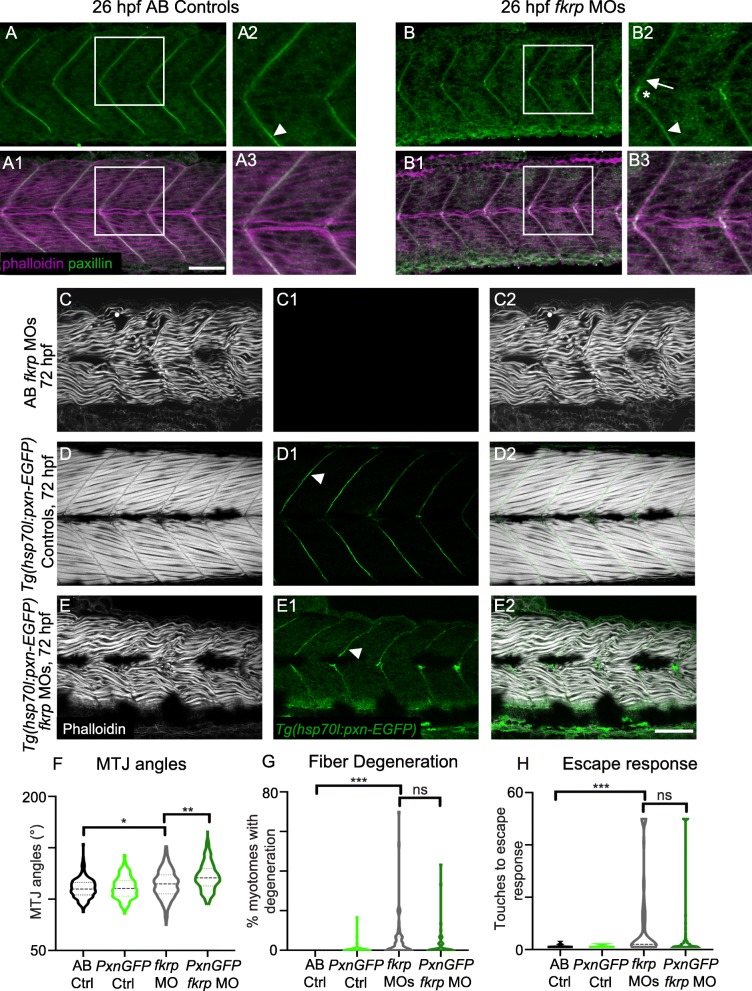
Paxillin overexpression does not improve muscle structure in *fkrp* morphants. (**A–B3**) Anterior left, dorsal top, side-mounted embryos at 26 hpf stained for Paxillin (green) and f-actin (purple). Paxillin concentrates at the MTJ (white arrowhead) in control embryos. There are some gaps in Paxillin localization in *fkrp* morphants (B2, white arrow) and Paxillin accumulates at the muscle pioneers (B2, white asterisk). (**C–E2**) Anterior left, dorsal top, side-mounted embryos at 72 hpf stained for f-actin (phalloidin, gray) in lettered and even numbered panels and expressing Paxillin-EGFP (green) in numbered panels. (**C–C2**) A *fkrp* morphant with disorganized muscle fibers. (**D–D2**) Control *Tg* (*hsp70l*:*pxna*-*EGFP*) embryo expressing Paxillin which concentrates at the MTJ (white arrowhead). (**E–E2**) *Tg* (*hsp70l*:*pxna*-*EGFP*) *fkrp* morphant. (**F**) MTJ angle quantification. *Fkrp* morphants with (*n* = 241 MTJs) and without (*n* = 170 MTJs) Paxillin-EGFP expression have a significant increase in average MTJ angle width compared with uninjected controls (*n* = 189, 191 MTJs). Paxillin overexpression in *fkrp* morphants significantly increases MTJ angles compared to morphants that do not overexpress Paxillin. (**G**) Fiber degeneration quantification. *Fkrp* morphants with (*n* = 55 embryos) and without (*n* = 60 embryos) Paxillin-EGFP expression have a significant increase in the percent of myotomes with degeneration per embryo compared to uninjected controls (*n* = 57, 54 embryos). Although there is a trend, Paxillin-overexpressing morphants do not have a significant reduction in dystrophy compared to control *fkrp* morphants. (**H**) Escape response quantification. *Fkrp* morphants with (*n* = 88 embryos) and without (*n* = 70 embryos) Paxillin-EGFP expression require significantly more touches to induce an escape response compared with uninjected controls (*n* = 66, 80 embryos). *Fkrp* morphants overexpressing Paxillin do not exhibit a significant improvement in escape response compared with *fkrp* morphants that do not overexpress Paxillin. Scalebars are 50 μm. *p < 0.05, **p < 0.01, ***p < 0.001, ns non-significant

Paxillin is an Integrin-associated protein that is required for cell adhesion to the ECM [74]. Paxillin concentrates at MTJs in wild-type muscle ([14], Fig. 3). Paxillin plays a role in the Nrk2b-NAD+-laminin cell adhesion pathway. Paxillin concentration at the MTJ is disrupted in *nrk2b* morphants and rescued with NAD+ [24]. Paxillin overexpression rescues *nrk2b* morphants [24]. Similarly, concentration of Paxillin at the MTJ is disrupted in *dag1* morphants and is improved with NAD+. Paxillin overexpression improves muscle structure in *dag1* morphants [25]. It is not known if either Paxillin concentration at the MTJ is disrupted in animal models of secondary dystroglycanopathy or if Paxillin overexpression improves muscle structure in these models. Paxillin concentration at the MTJ was disrupted in 26 hpf *fkrp* morphants (Fig. 3B) compared with controls (Fig. 3A). Paxillin does concentrate at the center of the MTJ adjacent to muscle pioneers that are slow-twitch fibers (Fig. 3B at the crux of the v-shaped MTJ, this was observed in 14/16 *fkrp* morphants). However, Paxillin concentration at the MTJ adjacent to fast-twitch muscle is disrupted (Fig. 3B2, arrow). These data suggest that Fkrp is required for normal development of the MTJ adjacent to fast-twitch muscle. Overexpression of Paxillin was sufficient for Paxillin to concentrate at the fast-muscle MTJ in *fkrp* morphants (Fig. 3E1 arrowhead). However, in contrast to what was previously observed in *dag1* morphants [25], Paxillin overexpression did not reduce muscle degeneration or improve the escape response in *fkrp* morphants (Fig. 3G-H). Paxillin overexpression actually increased MTJ angles (Fig. 3F). These data indicate that, in contrast to *dag1* morphants, Paxillin overexpression is not sufficient to improve muscle structure in *fkrp* morphants.

### NAD+ supplementation after initial muscle development improves MTJs but not muscle structure

**Fig. 4:**
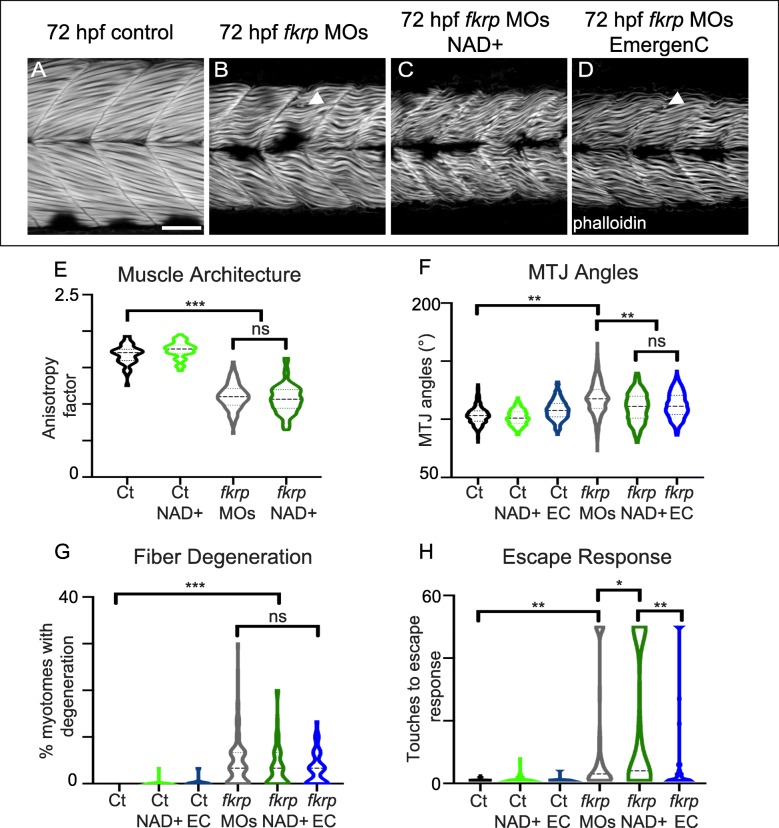
Later NAD+ supplementation improves MTJ structure, but NAD+ and EmergenC are required prior to initial muscle development to improve motility, fiber resilience, and fiber organization. (**a**–**d**) Anterior left, dorsal top, side-mounted embryos at 72 hpf stained for f-actin (phalloidin, gray). (**a**) Control embryo. (**b**) *fkrp* morphant. (**c**) *fkrp* morphant treated with NAD+ at 24 hpf. (**d**) *fkrp* morphant treated with EmergenC at 24 hpf. White arrowheads indicate single fiber detachments. (**e**) Fiber organization quantification. The anisotropy factor in embryos injected with *fkrp* MOs (*n* = 104 half-myotomes) is reduced compared to controls (*n* = 48 half-myotomes) and not improved with NAD+ supplementation at 24 hpf (*n* = 96 half-myotomes). (**f**) MTJ angle quantification. *Fkrp* morphants treated with NAD+ (*n* = 381 MTJs) or EmergenC (*n* = 318 MTJs) at 24 hpf have significantly decreased MTJ angles compared to untreated morphants (*n* = 651 MTJs). (**g**) Fiber detachment quantification. Although there is a trend towards reduced muscle degeneration, *fkrp* morphants receiving NAD+ (*n* = 55 embryos) or EmergenC (*n* = 41 embryos) at 24 hpf do not have a significant reduction in the percent of myotomes with dystrophy compared to untreated morphants (*n* = 98 embryos). (**h**) Escape response quantification. The number of touches required to induce an escape response is elevated in embryos injected with *fkrp* MOs (*n* = 128 embryos) compared to controls (*n* = 114 embryos). Morphants treated with NAD+ at 24 hpf (*n* = 81 embryos) have a worsened escape response compared to untreated morphants. Morphants treated with EmergenC at 24 hpf (*n* = 52 embryos) do not exhibit a significant change in escape response compared to untreated morphants, but significantly differ from NAD+-treated morphants. Scalebars are 50 μm. *p < 0.05, **p < 0.01, ***p < 0.001, ns non-significant

NAD+/EmergenC supplementation prior to muscle development is sufficient to improve muscle structure and function in *dag1* [25] and *fkrp* morphant embryos (Fig. 1). It is not known whether NAD+ or EmergenC can improve muscle structure and function *after* initial muscle development. We supplemented *fkrp* morphants with NAD+ or EmergenC at 24 hpf (after initial muscle development). Both NAD+ and EmergenC supplementation significantly improved MTJ angles (Fig. 4f). In contrast, neither NAD+ nor EmergenC supplementation at 24 hpf was sufficient to improve muscle fiber organization, reduce muscle degeneration (Fig. 4a–d, e, g), or improve the escape response (Fig. 4h). Thus, NAD+/EmergenC supplementation after initial muscle development is not sufficient to ameliorate muscle degeneration and improve muscle function in Fkrp-deficient zebrafish embryos.

### NMJ development is disrupted and partially improved with NAD+ supplementation in fkrp morphants

**Fig. 5:**
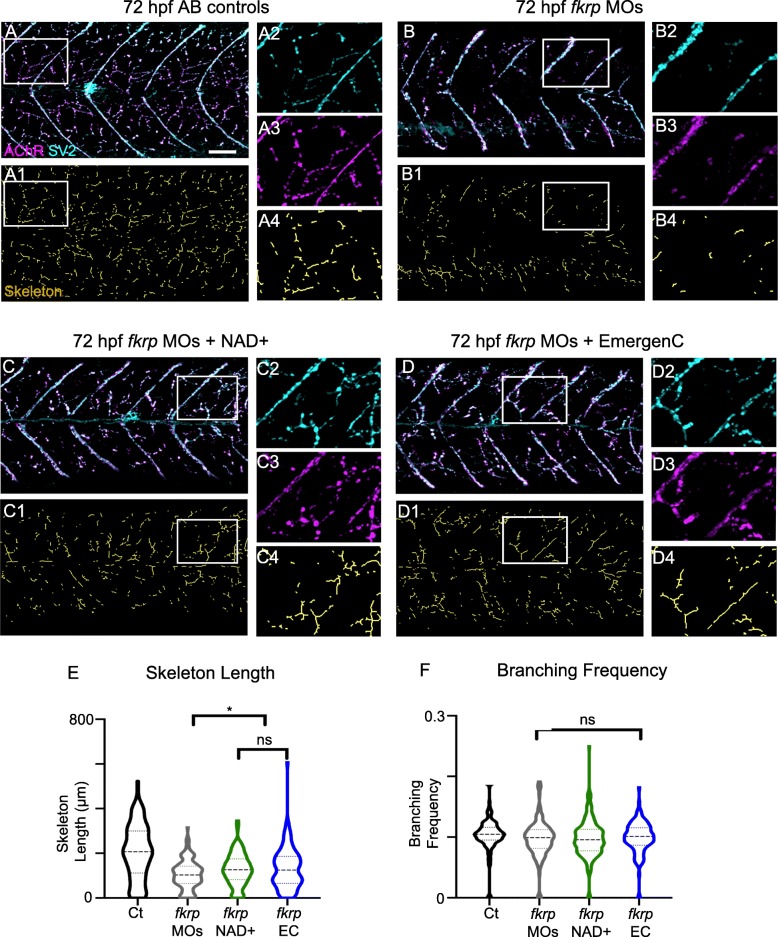
*Fkrp* morphants exhibit NMJ defects and NAD+ and EmergenC treatment prior to muscle development improves NMJ development. (**A–D4**) Anterior left, dorsal top, side-mounted embryos at 72 hpf with labeled AChR and SV2. (Lettered panels) Merged channels of AChR and SV2. (1) Skeletonized images. (2) Magnification of SV2 channel. (3) Magnification of AChR channel. (4) Magnification of skeleton channel. (**A–A4**) Control embryo. (**B–B4**) *fkrp* morphant embryo exhibiting reduced distributed innervation within the myotome. (**C–C4**) *fkrp* morphant embryo treated with NAD+ at 6 hpf shows increased NMJs. (**D–D4**) *fkrp* morphant embryo treated with EmergenC at 6 hpf also shows increased NMJs. (**E**) Length of skeletons. Skeleton length is reduced in *fkrp* morphants (*n* = 153 half-myotomes) compared to controls (*n* = 144 half-myotomes) but significantly increased in *fkrp* morphants receiving NAD+ (*n* = 183 half-myotomes) or EmergenC (*n* = 127 half-myotomes) at 6 hpf. (**F**) Degree of branching within the myotome in control embryos (*n* = 141 half-myotomes), *fkrp* morphants (*n* = 147 half-myotomes), and *fkrp* morphants receiving NAD+ (*n* = 179 half-myotomes) or EmergenC (*n* = 126 half-myotomes) at 6 hpf. Scalebars are 50 μm. *p < 0.05, **p < 0.01, ***p < 0.001, ns non-significant

The DGC is necessary for NMJ maturation after birth in mouse [32, 35, 43]. DG glycosylation is also necessary for normal NMJ formation during muscle regeneration [22]. It is generally thought that DG is not required for NMJ development but is required for NMJ maturation/stabilization/regeneration [27]. We asked whether initial NMJ development is disrupted in secondary dystroglycanopathies. NMJ structure at 72 hpf was analyzed by staining for SV2 and postsynaptic AChRs (alpha-bungarotoxin). We focused on analysis of innervation of fast-twitch fibers (distributed innervation, see skeletons Fig. 5). There is an extensive network of distributed NMJs in 72 hpf control embryos (Fig. 5A). This network is qualitatively disrupted in *fkrp* morphants where there appeared to be shorter chains of NMJs (Fig. 5B). Supplementation of NAD+ or EmergenC improved NMJ length, although NMJs were not fully restored (Fig. 5C, D). In order to quantify innervation, we developed a semi-automated technique in MATLAB to skeletonize NMJs (see methods). This analysis showed that although branching frequency was normal, skeleton length was reduced in *fkrp* morphants (Fig. 5E, F). NAD+ or EmergenC supplementation at 6 hpf slightly, but significantly, improved skeleton length in *fkrp* morphants (Fig. 5E). These results suggest that glycosylated DG is necessary for innervation, but not branching.

**Fig. 6:**
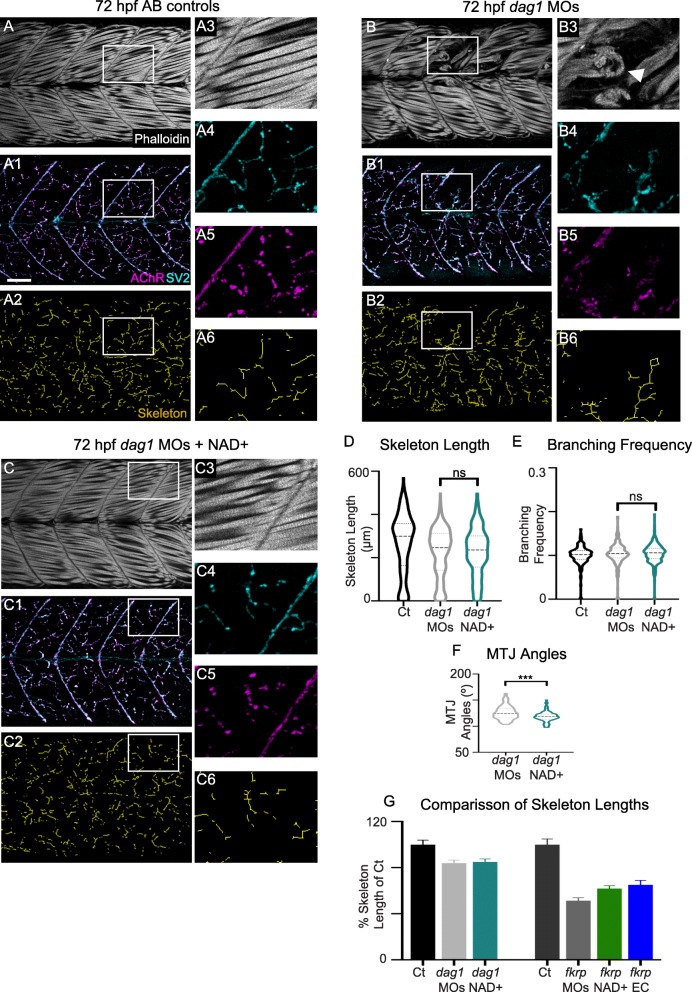
Skeleton length is less disrupted in *dag1* morphants than in *fkrp* morphants and is not significantly improved with NAD+ supplementation. (**A–C6**) Anterior left, dorsal top, side-mounted embryos at 72 hpf with labeled actin, AChRs, and SV2. (Lettered panels) Phalloidin stained embryos. (1) Merged channels of AChR and SV2. (2) Skeletonized images. (3) Magnification of phalloidin channel. (4) Magnification of SV2 channel. (5) Magnification of AChR channel. (6) Magnification of skeleton channel. (**A–A6**) Control embryo. (**B–B6**) *dag1* morphant embryo. (C–C6) *dag1* morphant embryo treated with NAD+ at 6 hpf. (**D**) Length of skeletons per myotome in control embryos (*n* = 186 half-myotomes), *dag1* morphants (*n* = 260 half-myotomes), and *dag1* morphants receiving NAD+ (*n* = 252 half-myotomes) at 6 hpf. Skeleton length is not significantly different between untreated and NAD+ treated *dag1* morphants. (**E**) Degree of branching within the myotome in control embryos (*n* = 180 half-myotomes), *dag1* morphants (*n* = 254 half-myotomes), and *dag1* morphants receiving NAD+ (*n* = 249 half-myotomes) at 6 hpf. NAD+ treatment made no significant difference. (**F**) MTJ angle quantification. MTJ angles are significantly reduced in *dag1* morphants receiving NAD+ (*n* = 98 MTJs) compared to untreated *dag1* morphants (*n* = 97 MTJs). (**G**) Bar graph of the percent skeleton length of control embryos per myotome. Note that the average percent skeleton length is more drastically reduced in *fkrp* morphants (51.4%, *n* = 153 half-myotomes) than in *dag1* morphants (83.9%, *n* = 260 half-myotomes). NAD+ supplementation increased this percentage in *fkrp* morphants (61.9%, *n* = 183 half-myotomes) but has little effect on *dag1* morphants (85.0%, *n* = 252 half-myotomes) Scalebars are 50 μm, error bars in (G) are standard error of the mean. *p < 0.05, **p < 0.01, ***p < 0.001, ns non-significant

As mentioned above, DG contributes to NMJ maturation and stabilization [27]. Given the above data showing that initial NMJ development is disrupted in *fkrp* morphants, we asked whether DG is also required for NMJ development. Skeleton length was slightly, but significantly, reduced in *dag1* morphants (Fig. 6B) compared to controls (Fig. 6A). However, NAD+ supplementation at gastrulation did not significantly increase skeleton length in *dag1* morphants despite rescuing other aspects of the phenotype (Fig. 6C, D, F). This result contrasts with what we observed in *fkrp* morphants supplemented with NAD+. Thus, we compared the relative severity of NMJ skeleton length defects in *fkrp* morphants versus *dag1* morphants. On average, *dag1* morphant skeleton length was 83.9% of that of uninjected controls. In contrast, *fkrp* morphant skeleton length on average was 51.4% of that of uninjected controls (Fig. 6G). Thus, NMJs are more severely disrupted in *fkrp* morphants compared to *dag1* morphants.

**Fig. 7:**
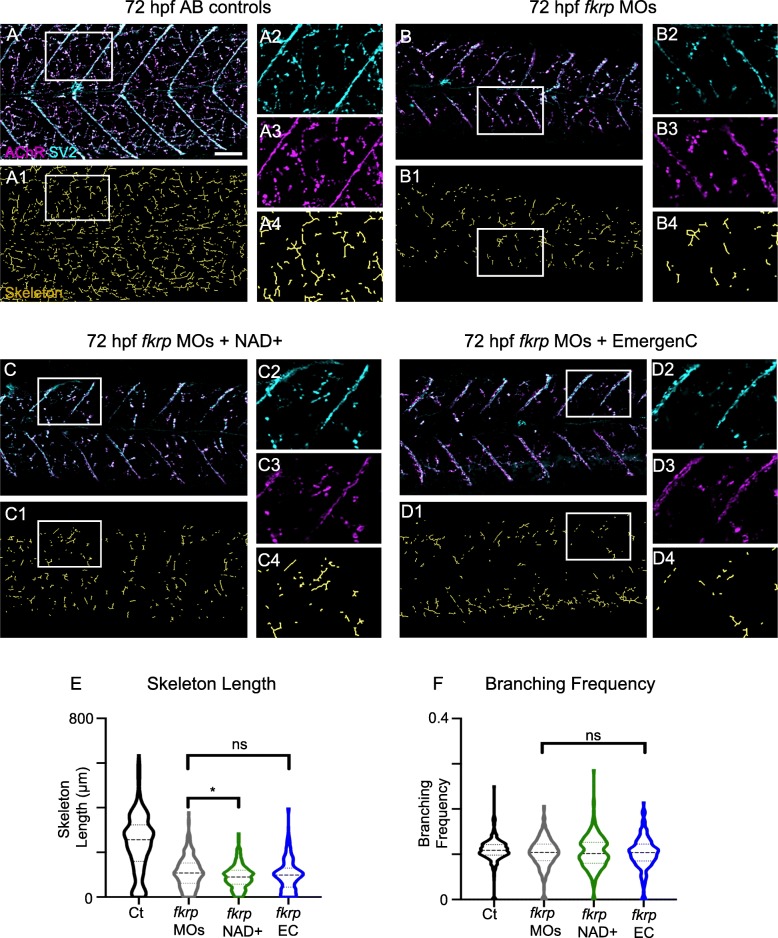
Supplementation with NAD+ or EmergenC after muscle development does not improve NMJ morphology in *fkrp* morphants. (**A-D4**) Anterior left, dorsal top, side-mounted embryos at 72 hpf with labeled AChRs and SV2. (Lettered panels) Merged channels of AChR and SV2. (1) Skeletonized images. (2) Magnification of SV2 channel. (3) Magnification of AChR channel. (4) Magnification of skeleton channel. (**A–A4**) Control embryo. (**B–B4**) *fkrp* morphant embryo exhibiting a reduced degree of distributed innervation within the myotome. (**C–C4**) *fkrp* morphant embryo treated with NAD+ at 24 hpf also has reduced innervation. (**D–D4**) *fkrp* morphant embryo treated with EmergenC at 24 hpf has reduced NMJs. (**E**) Length of skeletons per myotome in control embryos (*n* = 288 half-myotomes), *fkrp* morphants (*n* = 303 half-myotomes), and *fkrp* morphants receiving NAD+ (*n* = 225 half-myotomes) or EmergenC (*n* = 225 half-myotomes) at 24 hpf. Note that skeleton length is actually significantly decreased in *fkrp* morphants receiving NAD+ at 24 hpf. (**F**) Degree of branching within the myotome in control embryos (*n* = 283 half-myotomes), *fkrp* morphants (*n* = 295 half-myotomes), and *fkrp* morphants receiving NAD+ (*n* = 221 half-myotomes) or EmergenC (*n* = 210 half-myotomes) at 24 hpf. Scalebars are 50 μm. *p < 0.05, **p < 0.01, ***p < 0.001, ns non-significant

Somewhat surprisingly, we found that NAD+ supplementation at 24 hpf actually significantly worsened the escape response in *fkrp* morphants (Fig. 4, *p* < 0.05) despite the fact that we did not observe a significant deterioration of muscle structure. Thus, we asked how NAD+ supplementation at 24 hpf affected NMJ structure (Fig. 7). The effects of NAD+ on NMJ structure correlated with the motility defects: NAD+ at 24 hpf resulted in significantly shorter skeleton lengths (Fig. 7E). There was no significant difference between 24 hpf-treated and untreated morphants in terms of branching frequency (Fig. 7F).

### Muscle-specific expression of fkrp is not sufficient to rescue fkrp morphants

**Fig. 8:**
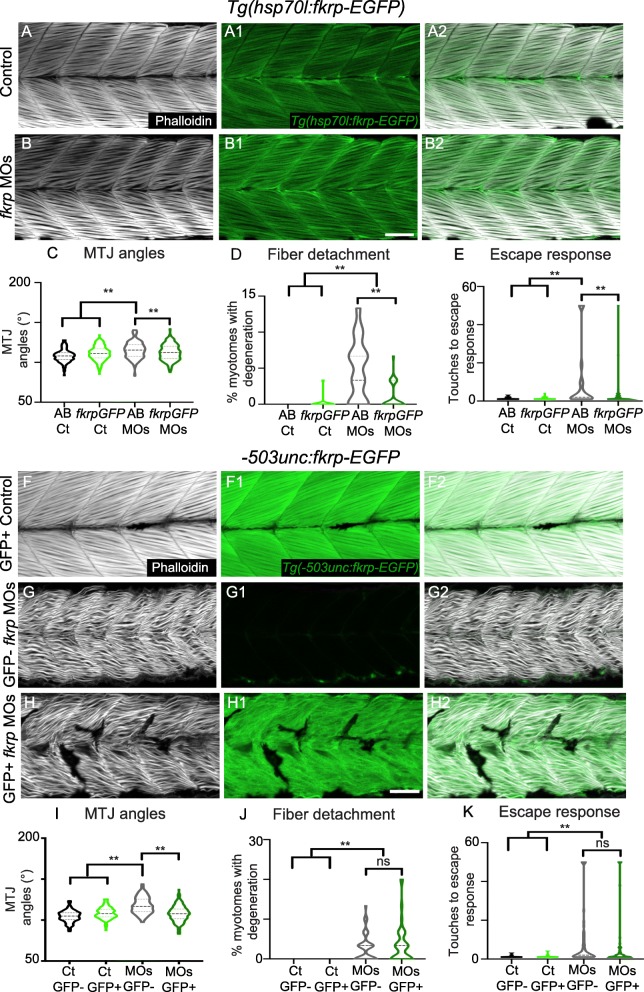
Muscle-specific overexpression of Fkrp improves MTJ angles, but not motility or fiber resiliency. (A–B2) Anterior left, dorsal top, side-mounted embryos at 72 hpf stained for f-actin (phalloidin, gray) and expressing Fkrp-EGFP (green). (A–A2) Control *Tg* (*hsp70l*:*fkrp*-*EGFP*) embryo expressing Fkrp in the fibers. (B–B2) *fkrp* morphant *Tg* (*hsp70l*:*fkrp*-*EGFP*) embryo expressing Fkrp in fibers. (C) MTJ angles of *Tg* (*hsp70l*:*fkrp*-*EGFP*) control (*n* = 264 MTJs) and *fkrp* morphant (*n* = 340 MTJs) embryos and AB controls and morphants (*n* = 141, 194 MTJs) at 72 hpf. Constitutive expression of Fkrp improves MTJ angles in morphants. (D) Constitutive expression of Fkrp in morphants (*n* = 72 embryos) significantly reduces the number of myotomes with fiber degeneration compared with control morphants (*n* = 35 embryos). (E) The escape response is significantly reduced in *fkrp* morphants constitutively overexpressing Fkrp (*n* = 82 embryos) compared to control morphants (*n* = 44 embryos). (F–H2) Anterior left, dorsal top, side-mounted embryos at 72 hpf stained for f-actin (phalloidin, gray) and expressing Fkrp-EGFP under control of the muscle-specific -*503unc* promoter (green). (F) Control *Tg*(*-503unc*:*fkrp*-*EGFP*) embryo expressing Fkrp specifically in muscle fibers. G) *fkrp* morphant embryo on *Tg*(*-503unc*:*fkrp*-*EGFP*) background lacking Fkrp expression in muscle fibers. (H) *fkrp* morphant *Tg*(*-503unc*:*fkrp*-*EGFP*) embryo expressing Fkrp specifically in muscle fibers does not show improved muscle organization. (I) Muscle specific expression of FKRP in *fkrp* morphants (*n* = 158 MTJs) significantly improves MTJ angles compared to control morphants (*n* = 153 MTJs). (J) Muscle-specific overexpression of Fkrp (*n* = 45 embryos) does not significantly lower the percent of myotomes with muscle degeneration in control *fkrp* morphants (*n* = 47 embryos). (K) There is not a significant difference in the number of touches required to induce an escape response in *fkrp* morphants that overexpress Fkrp (*n* = 73 embryos) in muscle versus those that do not (*n* = 79 embryos). Scalebars are 50 μm. *p < 0.05, **p < 0.01, ns non-significant

The above data indicate that Fkrp is necessary for early NMJ development and that disruption in NMJ morphology with 24 hpf NAD+ treatment correlates with worse motility. These results raise the possibility that Fkrp function is required in non-muscle tissues. We tested this hypothesis by expressing Fkrp in a muscle-specific manner. To confirm that constitutive Fkrp overexpression could rescue *fkrp* morphants, we injected *fkrp* MOs into a *Tg* (*hsp70l*:*fkrp*-*EGFP*) line we generated (Fig. 8A). Previous data suggest that overexpression of *fkrp* can be deleterious [77]. Although we observed a slight increase in MTJ angles (Fig. 8C), we did not observe any significantly adverse effects of Fkrp-EGFP on fiber degeneration or the escape response in control embryos (expression was induced at the 15 somite stage) (Fig. 8A, D, E). Global overexpression of Fkrp-EGFP in *fkrp* morphants improved fiber resiliency, MTJ angles, and the escape response compared to control morphants (Fig. 8B-E). These data indicate that (1) overexpression of Fkrp is not toxic under these conditions and (2) EGFP does not deleteriously affect Fkrp function. We next asked whether muscle-specific expression was sufficient to rescue the phenotype. We generated a transgenic line expressing Fkrp-EGFP under control of the -*503unc* promoter [5]. Muscle-specific overexpression of Fkrp did not affect muscle morphology in control embryos (Fig. 8F–F2). Muscle-specific overexpression of Fkrp in *fkrp* morphants decreased MTJ angles compared to EGFP negative control morphants (Fig. 8I). However, no other metrics of muscle structure/function were improved. Muscle-specific overexpression neither ameliorated fiber detachment (Fig. 8G, H, J) nor reduced touches required to induce an escape response (Fig. 8K). These data indicate that expression of Fkrp in muscle is not sufficient to rescue muscle morphology or function.

## Discussion

Dystroglycanopathies are a relatively understudied subset of muscular dystrophies. One aspect of dystroglycanopathies that is not well understood is how the myomatrix is impacted. Whether strategies that improve the myomatrix and muscle structure in a primary dystroglycanopathy (DG deficiency) are efficacious in secondary dystroglycanopathies are also not known. We previously showed that either NAD+ supplementation or overexpression of an Integrin-associated adaptor protein (Paxillin) are sufficient to improve muscle structure and function in DG-deficient zebrafish. Here, we show that muscle phenotypes in a zebrafish model of FKRP-associated dystroglycanopathy are improved with NAD+ supplementation but not Paxillin overexpression. Although the mechanisms are not known, these data clearly indicate that primary and secondary dystroglycanopathies show some differences. Our data also suggest that there are differences even within secondary dystroglycanopathies. Whereas muscle-specific expression of Large is sufficient to rescue muscle structure and function in *Large*/*myd* mutant mice [27], we found that muscle-specific expression of *fkrp* is not sufficient to improve muscle structure/function in *fkrp* morphant zebrafish. Finally, our data show that NMJ formation is disrupted earlier in development than has previously been observed in a primary and secondary dystroglycanopathy model. Taken together, these data indicate that it is necessary to study the secondary dystroglycanopathies individually within the context of the group as a whole.

### Cell-matrix adhesion and secondary dystroglycanopathy

Adhesion of muscle fibers to the MTJ is necessary for muscle development and homeostasis. We previously identified a cell adhesion pathway that contributes to laminin organization at the MTJ: Nrk2b-mediated NAD+ production potentiates laminin organization at the MTJ during zebrafish muscle development [24]. We then demonstrated that exogenous NAD+ is sufficient to improve laminin organization, muscle structure, and muscle function in zebrafish deficient for either DG or Itga7 [25]. DG and Itga7 are both transmembrane receptors for laminin. We hypothesized that NAD+ improved laminin organization, at least in part, by increasing clustering of the remaining receptor (DG in Itga7-deficient embryos, and Itga7 in DG-deficient embryos). Here, we asked whether laminin organization would be increased if DG is present but hypoglycosylated. The rationale was that it is possible that the presence of hypoglycosylated DG that cannot bind laminin could inhibit the improvement of laminin organization with NAD+. The ribitol 5-phosphate transferase FKRP is necessary for glycosylation of DG [21, 38]. We asked if NAD+ was sufficient to improve cell-matrix adhesion in *fkrp* morphant zebrafish embryos. NAD+ supplementation prior to muscle development was sufficient to improve laminin polymerization, muscle structure, and the escape response.

Organization of laminin at the MTJ is disrupted in zebrafish deficient either for DG [25] or Fkrp [39, 47, 72] (Fig. 1). Laminin is required for concentration of the Integrin adaptor protein Paxillin to the MTJ [24]. Paxillin is an intracellular protein that localizes to cell-ECM adhesion complexes [15] and modulates ECM composition at the developing MTJ [34]. Previous data regarding the beneficial potential of Paxillin in the context of aberrant muscle development/homeostasis are contradictory. Paxillin overexpression worsens muscle damage in ethanol-treated zebrafish [10]. However, Paxillin overexpression is sufficient to improve MTJ morphology in Nrk2b-deficient zebrafish [24]. Paxillin overexpression also improves laminin organization and reduces muscle degeneration in *dag1* morphant zebrafish [25]. We hypothesized that Paxillin overexpression would improve muscle structure and function in *fkrp* morphants where DG is present but not properly glycosylated. Interestingly, we found that Paxillin expression trended towards reducing degeneration and improving the escape response, but the effects were not significant (Fig. 3). This result suggests the hypothesis that, in contrast to the situation where DG is absent, the presence of hypoglycosylated DG in the membrane prevents Paxillin-mediated stabilization of muscle fibers.

### Benefits of NAD+ and EmergenC supplementation may be restricted to the neuromusculoskeletal system

Both *fkrp* morphants and mutants have an increased UPR. X*pb1*, a marker of the UPR, is upregulated in both *fkrp* morphants and *fkrp* mutants [47, 63]. Expression of *bip*, a marker of the UPR, is upregulated in *fkrp* morphants at 28 hpf, especially in the neural floor plate and the hatching gland [47]. However, *glytl1b* (*large2*) morphants with hypoglycosylated DG, *dag1* morphants, and *sly/lam1c* mutants do not exhibit *bip* upregulation [47], suggesting that activation of the UPR may result directly from loss of Fkrp. We asked if EmergenC supplementation was sufficient to reduce the UPR in *fkrp* morphants. We found that 3 dpf *Tg* (*ef1α*:*xbp1δ*-*GFP*) embryos injected with *fkrp* MOs have increased *xbp1*-*GFP* fluorescence compared to control embryos. EmergenC supplementation at gastrulation was not sufficient to reduce *xbp1*-*GFP* fluorescence. Taken together, these data suggest that activation of the UPR occurs independently of muscle-cell matrix adhesion and DG glycosylation and is likely a direct consequence of Fkrp knockdown.

*Fkrp* morphants and *fkrp* mutants also exhibit aberrant vascular development, including reduced ISV lengths [63, 78]. One study suggests that impaired vascularization directly results from loss of Fkrp and that muscle phenotypes and vascular phenotypes are independent of one another [78]. Here, we demonstrate that NAD+ supplementation at gastrulation is not sufficient to rescue truncated ISVs in *fkrp* morphants. Given that NAD+ improves muscle structure and function in *fkrp* morphants, our data suggest that vascular phenotypes observed in *fkrp* morphants are not dependent on muscle phenotypes. This supports the previous hypothesis that abnormal vascular development in zebrafish models of FKRP-associated dystroglycanopathy is a direct result of loss of Fkrp.

### Timing of NAD+ intervention is critical

Gene therapy [55, 71, 73, 77], estrogen receptor modulators [79], and exogenous ribitol supplementation [9] improve muscle structure in FKRP-associated dystroglycanopathy models. The efficacy of adenoviral gene therapy and ribitol supplementation decreases if administered later in the mouse’s lifespan [9, 77], suggesting that early intervention is most beneficial. Our data regarding the timing of NAD+ and EmergenC supplementation in *fkrp* morphants are consistent with the data mentioned above. We found that NAD+ (or EmergenC) supplementation prior to muscle development increased laminin organization, reduced muscle degeneration, improved muscle function, and improved NMJ structure (Fig. 1). In contrast, NAD+ (or EmergenC) supplementation after initial muscle development only improved MTJs and not muscle structure/function (Fig. 4).

Our data do not resolve why MTJ morphology is improved with late NAD+ supplementation or muscle-specific overexpression of Fkrp. Improved MTJ morphology does not always correlate with fiber degeneration: *itga7* morphants exhibit muscle fiber detachment but have normal MTJ morphology. NAD+ supplementation improves MTJ angles, but not fiber degeneration in *dag1/itga7* double morphants [25]. Movement is not necessary for MTJ chevron formation in zebrafish embryos [59]. We show that motility is not improved in *fkrp* morphants receiving NAD+ or EmergenC after initial muscle development, further suggesting that improved MTJ morphology does not always correlate with mobility.

While this manuscript was in preparation, it was shown that administration of pentetic acid at 48 hpf improved muscle and pericardiac phenotypes in a *fkrp* mutant zebrafish model of LGMD2I [63]. Pentetic acid is a chelating agent that binds Ca2+ and Mg2+. The mechanism by which this improves muscle phenotypes is currently unknown. However, abnormal Ca2+ levels have been implicated in DMD and inducing an influx of Ca2+ is sufficient to induce dystrophy [50]. Together, these data suggest that different therapeutic avenues have different time windows of efficacy in multiple animal models.

### Dag1 and fkrp are required for proper NMJ development

NMJ development requires orchestrated interactions between muscle cells and motor neurons. The DGC is a major component of NMJs. Thus, it is not surprising that NMJ morphology is disrupted in multiple dystroglycanopathies. Fukutin-deficient chimeric mice have abnormal AChR clustering and NMJs at postnatal day 15 [61]. Newborn pups homozygous for *Large/myd* mutations have altered NMJs as well [32]. These data clearly indicate that NMJs are abnormal after birth. What is not clear is at which point in embryonic development NMJ disruption occurs. Do NMJs develop normally and then degenerate or is initial NMJ development abnormal? Here, we provide evidence that early NMJ development is slightly disrupted in DG-deficient zebrafish, with NMJ chains that innervate fast-twitch muscle being approximately 84% of control chain length. To our knowledge, this is the earliest developmental stage at which NMJ disruption has been observed in a primary or secondary dystroglycanopathy. Interestingly, *fkrp* morphants exhibit more dramatic disruption of NMJ morphology, with NMJ chains about half of control chain length. We do not know the mechanisms underlying the more severe disruption in NMJ development in *fkrp* versus *dag1* morphants. One possibility is that hypoglycosylated *dag1* acts as a “dominant negative” and thus disrupts NMJ development more than when *dag1* is not present. Additionally, there are many glycosylated proteins at the NMJ, such as Lrp4, Musk, and Agrin. Thus, the more severe disruption in *fkrp* morphants could also reflect a requirement of Fkrp for glycosylation of other NMJ resident proteins.

### Muscle-specific overexpression of Fkrp is not sufficient to rescue fkrp morphants

Overexpression of LARGE is sufficient to synthesize glycan-enriched alpha-DG and is sufficient to improve laminin-binding activity in multiple dystroglycanopathy patient cell lines [3]. Muscle-specific expression of *Large* is sufficient to rescue muscle structure, neurotransmission, and NMJs in *Large/myd* mutant mice [27]. NMJ defects in *Large/myd* mutant mice were observed after birth [32]. The above data suggest that, in the context of Large-associated dystroglycanopathy, NMJ defects are secondary to muscle disruption. Given that we observed very early developmental disruption of NMJs, we could not necessarily conclude that NMJ disruption in Fkrp-deficient embryos is secondary to the muscle phenotype. Thus, we tested whether muscle-specific expression of Fkrp would be sufficient to rescue neuromuscular function in *fkrp* morphant embryos. We first showed that global expression of Fkrp (under control of the heat shock promoter) is sufficient to rescue *fkrp* morphant embryos. This result indicates that the Fkrp-EGFP fusion protein is functional.

Next, we expressed Fkrp in muscle under control of the muscle specific -*503unc* promoter [5]. Muscle-specific expression of Fkrp was sufficient to slightly, but significantly, reduce MTJ angles. However, muscle-specific expression of Fkrp was not sufficient to reduce muscle degeneration or improve the escape response (Fig. 8). We do not know why our results differ from the Large study. We do not believe that the timing of expression is the key factor because muscle creatine kinase (Large study) and Unc45b (our study) are expressed at similar times during myotome development. In the DG glycosylation pathway, FKRP works in concert with fukutin (FKTN) to synthesize a tandem ribitol phosphate that connects a core o-linked glycan with matriglycan, the ligand for laminin and other ECM proteins [38]. This step precedes the completion of matriglycan synthesis by LARGE. Roles for LARGE or FKRP in other glycosylation reactions have not been identified. However, the fact that muscle-specific expression of Fkrp is not sufficient to rescue muscle homeostasis may suggest that Fkrp is required to glycosylate proteins other than DG. Regardless, our data indicate that Fkrp is required outside of skeletal muscle to reduce muscle degeneration. In the future, it will be interesting to determine in which tissues and cells Fkrp is required to rescue neuromuscular function in Fkrp-deficient embryos.

## Conclusion

The myomatrix is critical for muscle development and homeostasis and is disrupted in many dystroglycanopathies studied thus far [17, 47, 48, 58, 68]. Here, we primarily focused on two specialized junctions within the myomatrix: the MTJ and NMJ. MTJ and NMJ development are disrupted in *fkrp* morphants. NAD+ supplementation prior to muscle development, which improves myomatrix organization in DG-deficient zebrafish, was sufficient to improve muscle homeostasis, MTJ structure, and NMJ formation in *fkrp* morphant zebrafish. However, NAD+ did not improve vascular or UPR phenotypes in *fkrp* morphants, and NAD+ supplementation after initial muscle development was not particularly efficacious. There was a slight improvement in MTJ structure and a reduced variation in muscle degeneration. These data are similar to previous studies showing that earlier intervention has improved outcomes in *FKRP* mutant mice [77]. Interestingly, in contrast to muscle-specific expression of Large in *Large/myd* mutant mice, muscle-specific overexpression of Fkrp was not sufficient to improve muscle structure/function in *fkrp* morphants. Taken together, our data indicate that Fkrp plays an early and crucial role in muscle, MTJ, and NMJ development.

## Additional file

Additional file 1 : Figure S1 Scoring of relative MTJ staining intensity. (A–C) Anterior left, dorsal top, side-mounted zebrafish embryos at 26 hpf stained for laminin-111 at the MTJ (purple). (A) Example of an embryo with strong laminin staining intensity at the MTJ. (B) An embryo with weak laminin staining intensity at the MTJ. (C) An embryo lacking laminin staining at the MTJ. (EPS 1671 kb)

## Data Availability

All detailed statistical analyses and a compilation of all images will be uploaded to figshare upon acceptance.

## Abbreviations

AChR: Acetylcholine receptor
DG: Dystroglycan
DGC: Dystrophin-glycoprotein complex
DMD: Duchenne Muscular Dystrophy
dpf: Days post-fertilization
ECM: Extracellular matrix
ER: Endoplasmic reticulum
ERM: Embryo rearing medium
FKRP: Fukutin-related protein
GFP: Green fluorescent protein
GMPPB: GDP-mannose Pyrophosphorylase B
hpf: Hours post-fertilization
ISPD: Isoprenoid Synthase Domain-Containing
ISV: Intersegmental vessel
Itga7: Integrin alpha7
LGMD2I: Limb-Girdle Muscular Dystrophy Type 2I
MO: Morpholino
MTJ: Myotendinous junction
NMJ: Neuromuscular junction
NAD+: Nicotinamide riboside
Nrk2b: Nicotinamide riboside kinase 2b
PFA: Paraformaldehyde
SV2: Synaptic vesicle protein 2
Tg: Transgenic
UPR: Unfolded protein response
